# Invited Review: Glucosinolates Might Result in Low Methane Emissions From Ruminants Fed Brassica Forages

**DOI:** 10.3389/fvets.2020.588051

**Published:** 2020-10-09

**Authors:** Xuezhao Sun

**Affiliations:** ^1^The Innovation Center of Ruminant Precision Nutrition and Smart and Ecological Farming, Jilin Agricultural Science and Technology University, Jilin City, China; ^2^Jilin Inter-regional Cooperation Center for the Scientific and Technological Innovation of Ruminant Precision Nutrition and Smart and Ecological Farming, Jilin City, China

**Keywords:** digesta retention time, free triiodothyronine, greenhouse gas, physiological parameters, plant secondary compounds, rumen

## Abstract

Methane is formed from the microbial degradation of feeds in the digestive tract in ruminants. Methane emissions from ruminants not only result in a loss of feed energy but also contribute to global warming. Previous studies showed that brassica forages, such as forage rape, lead to less methane emitted per unit of dry matter intake than grass-based forages. Differences in rumen pH are proposed to partly explain these low emissions. Rumen microbial community differences are also observed, but the causes of these are unknown, although altered digesta flow has been proposed. This paper proposes a new mechanism underlying the lower methane emissions from sheep fed brassica forages. It is reported that feeding brassica forages to sheep can increase the concentration of free triiodothyronine (FT_3_) in serum, while the intramuscular injection of FT_3_ into sheep can reduce the mean retention time of digesta in the rumen. The short retention time of digesta is associated with low methane production. Glucosinolates (GSLs) are chemical components widely present in plants of the genus *Brassica*. After ruminants consume brassica forages, GSLs are broken down in the rumen. We hypothesize that GSLs or their breakdown products are absorbed into the blood and then may stimulate the secretion of thyroid hormone FT_3_ in ruminants, and the altered thyroid hormone concentration may change rumen physiology. As a consequence, the mean retention time of digesta in the rumen would be altered, resulting in a decrease in methane emissions. This hypothesis on mitigation mechanism is based on the manipulation of animal physiological parameters, which, if proven, will then support the expansion of this research area.

## Introduction

Methane (CH_4_) is an important greenhouse gas, with a global warming potential of 28 times more than carbon dioxide (1). Agriculture accounts for 62% of CH_4_ emissions from anthropogenic activities, while ruminants account for 58% of the CH_4_ emissions from agriculture (2). As a result, enteric CH_4_ emissions are the single largest source of anthropogenic CH_4_ contributing to the global greenhouse gas emissions (3). Methane emissions also cause energy losses in livestock, which account for 3.9–10.7% of the metabolic energy ingested, resulting in less efficient energy utilization by the animal (4). Reducing CH_4_ emissions, therefore, has the potential to improve feed conversion efficiency (5). Thus, mitigation of CH_4_ emissions helps not only environmental protection but also has substantial economic benefits to promote sustainable development of animal husbandry (6, 7). For this reason, mitigation of CH_4_ emissions from ruminants has become a highly active research topic in animal husbandry.

Methane is formed in the process of the rumen microbial degradation of feed in ruminants. The approaches to the mitigation of CH_4_ emissions include inhibiting methanogens with inhibitors (8) or a vaccine, modifying microbial activity in the rumen with electron acceptors, ionophores (9), or dietary manipulation (10), and breeding for low-CH_4_ livestock (11). Among these approaches, the use of brassica forages to mitigate CH_4_ emissions is a feasible method that does not change farming systems, increase production costs or result in artificial chemical residues.

The purpose of this review is to summarize literature reports on the use of brassica forage to mitigate CH_4_ emissions, analyze possible mitigation mechanisms, and highlight the possible role of glucosinolates, which are characteristic substances in brassica forages, in reducing CH_4_ emissions.

## Ruminants Fed Forage Brassica Emit Low Methane

Brassica forage crops including kale (*Brassica oleracea*), turnip (*Brassica campestris*), forage rape (*Brassica napus*), and swede (*Brassica napus* ssp. *rapifera*) are annual plants, grown worldwide to provide ruminants feed, in many cases during the period when forage supply is limited in quantity or quality (12). These crops can grow in winter, but forage rape and bulb and leafy turnips can also grow in summer. These crops have high water-use efficiency and thus are suitable to grow in conditions of limited water resources (13). They have a short growth period (14), being easy to grow, with the ability to be intercropped with legumes (15).

Forage brassica crops have the characteristics of having a high yield, typically 2–8 t dry matter (DM)/ha for leafy turnip, 3–10 t/ha for forage rape, 2–12 t/ha for turnips, 5–20 t/ha for kale and 5–20 t/ha for swedes (16). The leaves and stems of kale, leafy turnip, and forage rape are used for feed, while swede and bulb turnip are root brassicas with both leaves and bulbs being used. The chemical composition of brassica forages varies greatly among species and within a species mainly due to the difference in the ratio of leaves to bulbs or to stems (17). Compared with perennial ryegrass (*Lolium perenne*), brassica forages contain less neutral detergent fiber (NDF) and more readily fermentable carbohydrate (12, 18). The content of NDF is 271–328 g/kg DM for kale, 180–240 g/kg DM for forage rape and turnip, and 165–196 g/kg DM for swede (12, 17, 18, 18–20), while the content of readily fermentable carbohydrates was 253 g/kg DM for kale, 285 g/kg DM for forage rape, 334 g/kg DM for turnip, and 370 g/kg DM for swede (18). Among readily fermentable carbohydrates, the content of pectin is about 69–94 g/kg DM (18), the content of sugars (raffinose, sucrose, glucose, fructose) and starch is 205 g/kg DM for kale, 138 g/kg DM for forage rape, 194 g/kg DM for turnip, and 283 g/kg DM for swede (17). The content of crude protein is 130–162 g/kg DM for bulb brassicas and 167–193 g/kg DM for leafy brassicas (18). The low content of NDF and the high content of readily fermentable carbohydrates lead to a high ruminal degradation rate (21, 22), a high DM digestibility (810–890 g/kg), and a high metabolizable energy content (12.1–14.1 MJ/kg DM) and thus have a high feeding value for ruminants (12, 18). As a result, these crops have been applied in farming practice in sheep (23, 24), beef cattle (25), dairy cows (25–27), and deer (12).

Research on the use of brassica forages for the mitigation of CH_4_ emissions from ruminants began in New Zealand. Sun et al. (18) reported for the first time that four common forage brassica crops in New Zealand kale, turnip, rape, and swede fed to sheep in winter resulted in lower CH_4_ yield (CH_4_ emissions per unit of DM intake) by 10, 6, 25, and 23%, respectively, compared with the control perennial ryegrass.

A series of animal experiments were conducted after this study (28). These experiments were conducted under various conditions including short- vs. long-term feeding (29), indoor feeding vs. grazing (30), winter vs. summer varieties (31), different brassica types (32), and primary growth vs. regrowth (32). Under these conditions, CH_4_ emissions were always lower than the control perennial ryegrass-based pasture. When forage rape was mixed with perennial ryegrass to form mixed diets with gradual inclusion levels for sheep, CH_4_ yields declined linearly with the increase in the proportion of forage rape in the diet (33). Heifers fed forage rape also emitted less CH_4_ than those fed perennial ryegrass-based pasture (34). A study conducted in Australia showed that feeding dairy cows with brassica forage (*B. napus* cv. *Winfred*) during summer resulted in a 21% lower CH_4_ yield than feeding chicory (*Cichorium intybus*) (35). It was concluded that both sheep and cattle fed different forage brassica crops in different seasons as a sole diet, or as a component of a mixed diet, under housed feeding or grazing conditions, emit low CH_4_ to varying degrees, and the mitigation effect does not disappear with extended feeding.

## Secondary Metabolites in Forage Brassica May Contribute to Low Methane Emissions

The known mechanisms for the mitigation of enteric CH_4_ emissions mainly include the manipulation of rumen microbiota by methods such as the addition of CH_4_ inhibitors, and the manipulation of fermentation substrates of rumen microorganisms, such as altering dietary composition and providing electron acceptors (10, 36). These mechanisms cannot fully explain the low CH_4_ emissions with forage brassicas.

Lower CH_4_ yields from forage brassica were associated with differences in the rumen microbiota compared to perennial ryegrass, with a proposal for shifts in fermentation to more propionate and less hydrogen, resulting in less CH_4_ (29). However, the factors resulting in this rumen microbial community difference are not fully understood. A multiple regression analysis of the conventional nutrients of brassica crops and CH_4_ yield showed that only water content among these nutrients had a weak correlation with emissions (37). Nitrate and sulfate can be used as electron acceptors to reduce CH_4_ emissions (38), but their contents in forage brassica crops vary widely (12, 39). Even the highest contents in brassica crops explain but a small proportion of the reduction in CH_4_ emissions (18, 29). A meta-analysis of the relationship between rumen fermentation parameters and CH_4_ yield in sheep showed that fermentation type, as indicated by the ratio of acetate to propionate and butyrate, had a limited effect (40). A low rumen pH is associated with low methanogenesis (41, 42). While the rumen pH was low in sheep fed forage rape (29), a study in which rumen pH of forage rape-fed sheep was manipulated by adding sodium carbonate did not suggest that the low CH_4_ yield results totally from low rumen pH (43). The rumen microbial community of sheep fed forage rape differed greatly from that of sheep fed perennial ryegrass. For example, there were increased abundances of *Selenomonas* and *Sharpea*, whereas *Selenomonas* is known to produce acetate and propionate or lactate and *Sharpea* are lactate producers (44) and linked to low CH_4_ via a proposed pathway (45). However, the importance of altered microbial community in low CH_4_ emissions might be limited (29). In summary, although rumen fermentation type has a limited effect, and some factors such as conventional nutrients, nitrate and sulfate in forage brassica can be ruled out for the explanation of low CH_4_ emissions with forage brassica, the reason why is still unclear.

It has been reported that chicory (46, 47) and white clover (*Trifolium repens*) (48) do not result in lower CH_4_ emissions compared with perennial ryegrass-based pasture. Chicory, white clover and forage brassicas are dicotyledonous plants. The conventional nutritional composition is similar, but the secondary metabolites between them are different. The discrepancy in CH_4_ emissions from these plants suggests that secondary metabolites in forage brassicas might play a role in mitigation. Glucosinolates (GSLs) and *S*-methyl-L-cysteine sulfoxide (SMCO) are two types of plant secondary metabolites which are widely present in *Brassica* plants (49, 50). Therefore, these two types of compounds should be a focus for exploring the mechanisms underlying the low CH_4_ emissions with brassica forages.

## Secondary Metabolites in Forage Brassicas

**Figure 1:**
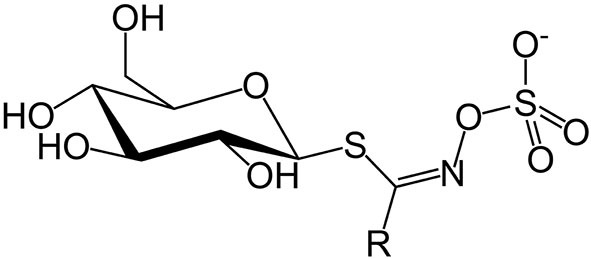
Glucosinolate structure (the side group R varies).

Concerns over GSLs and SMCO mainly due to anti-nutritional effects in animals (12, 51) and possible beneficial effects to human health (52, 53) contributed to the need for this review. Glucosinolates are a class of sulfur-containing anionic hydrophilic plant secondary metabolites whose core structure is β-D-glucose linked to a sulfonate aldoxime group (Figure 1), and a side chain derived from amino acids (54).

**Figure 2:**
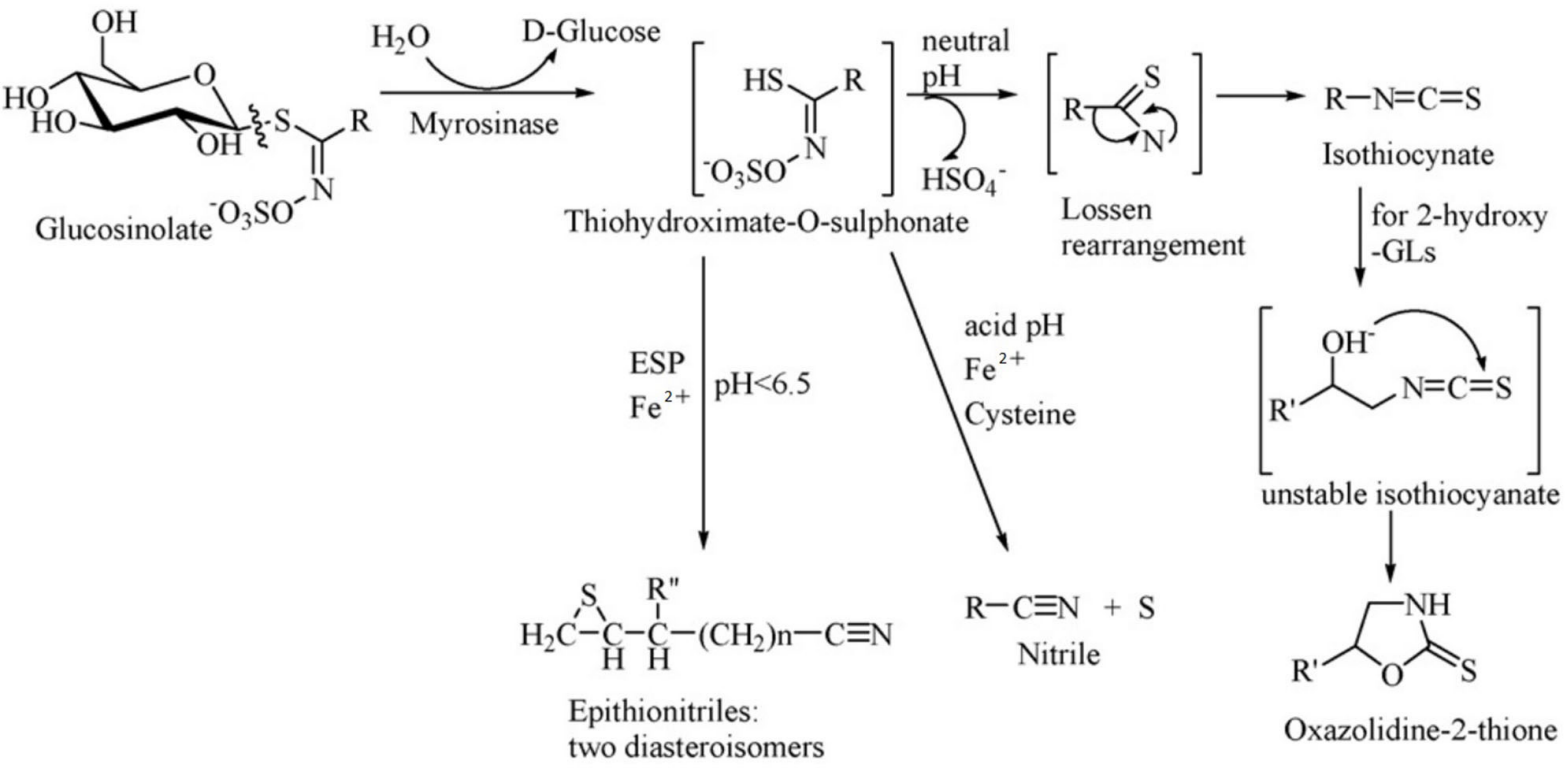
Diagram of enzymatic hydrolysis of glucosinolates. Adapted from Tripathi and Mishra (56).

Glucosinolates have no physiological activity *per se*, and after combination with β-glucosidase (also known as myrosinase), they are degraded to a variety of biologically active substances, which have toxic effects on herbivores and also function as repellents (55). Glucosinolates and β-glucosidase are present in different cells or different areas of the same cell in plants, and they react when the plant is mechanically damaged or chewed by the animal. The products of the breakdown are mainly isothiocyanate (ITC), thiocyanate, nitrile, epithionitrile and oxzolidine-2-thione, of which ITC is the most important product (Figure 2) (52).

There are extensive studies of GSLs in brassica vegetables and oil crops (57), but there are few studies on brassica forages. Velasco et al. (58) found that GSL profile differs greatly in rape varieties for the use as vegetables, oilseeds and feeds. Knowledge gained from brassicas for other uses cannot be applied directly to brassica crops for feed use. It has been reported that there are many types of GSLs in brassica forages, up to 18, but the contents of individual GSLs vary greatly (Table 1). Among them, 3–4 GSLs are predominant, accounting for more than 80% of the total content. Each brassica forage crop has its own predominant GSLs, but glucobrassicanapin is generally more than 40% of the total GSL content in forage rape, swede and turnip, and sinigrin exceeds 40% of the total GSL content in kale (18). The breakdown products of GSLs in brassica forages have been reported (59) and knowledge about them is generally derived from research results from brassica crops used for other purposes (52). Rapeseed cake contains a large amount of GSLs and the structure, breakdown products and their effects on animals have been extensively reviewed (56). Glucosinolates produce mainly ITC and nitriles in the rumen. During digestion, about 21–41% of GSLs in kale leaves are converted to nitriles, 37% for swede leaves and 50% for swede bulbs (12). Nitriles are not degraded for at least 23 h after sheep consume kale, but completely degraded within 4 h for swede bulbs and leaves.

**Table 1:** Concentration of total glucosinolates (GSLs) and proportion of individual GSLs in brassica forages^a^.

**Items**	**Kale (*Brassica oleracea*)**	**Rape (*B. napus*)**	**Swede (*B. napus*)**	**Turnip (*B. campestris*)**
Total glucosinolate(μmol/kg dry matter)	229.3	308.4	803.8	1218.1
Proportion of individual GSLs in the total GSLs				
Sinigrin	40.6	0.0	0.0	0.0
Glucobrassicanapin	0.1	44.4	40.0	44.8
Epiprogoitrin	23.6	17.7	16.9	13.1
Gluconapin	17.5	10.5	14.0	22.2
Gluconasturtiin	0.8	3.6	14.6	6.9
Gluconapoleiferin	0.0	8.3	4.9	7.1
Glucoraphanin	8.0	0.7	0.8	0.3
Glucobrassicin	7.4	5.4	0.2	0.2
Glucoalyssin	0.3	4.7	2.4	1.0
Progoitrin	0.0	3.2	3.0	3.3
Sinalbin	1.7	1.5	0.7	0.4
Glucoiberin	0.0	0.0	1.6	0.4
Glucoerucin	nd^b^	nd	0.9	0.2
Glucotropaeolin	0.0	0.0	0.0	0.0
4-hydroxyglucobrassicin	0.0	nd	0.0	0.0
Glucobarbarin	nd	nd	0.0	0.0
Glucoraphenin	nd	nd	0.0	nd
Glucosibarin	nd	nd	0.0	nd

a *Adapted from Sun et al. (18)*.

b *nd, not detected*.

The non-protein amino acid SMCO is about 1–2% by dry weight in brassica plants (60). The contents of SMCO vary among forage brassica species and is especially high in kale (12). The SMCO contents are also affected by fertilizer application (12), silage making (61) and harvesting (62). When plant tissues are broken, cysteine sulfoxide lyases in the vacuole are released, resulting in decomposition of SMCO into ammonia, pyruvate and methanesulphenic acid (60). Complete conversion of SMCO to dimethyl disulphide occurs in the rumen, and dimethyl disulphide inactivates proteins by combining the sulphdryl group in proteins. For example, dimethyl disulphide can reduce the content of hemoglobin, and even cause anemia, and can also affect the production of host and microbial proteins in the body. *S*-methyl-L-cysteine sulfoxide can increase ghrelin and thyroid hormones in the plasma, which can stimulate the body's protein synthesis to replace the protein inactivated by dimethyl sulfoxide (12).

## Secondary Metabolites Might not Directly Inhibit Methanogens

Jayanegara et al. (63) found that *Brassica crassifolia* resulted in 25% less CH_4_ emissions than grass hay in an *in vitro* batch culture study. Dillard et al. (64) studied the methanogenesis of brassica forage crops using a continuous fermentation system with half of the culture substrate as *Dactylis glomerata* and the other half as forage rape, oilseed rape (*B. napus*), turnip or annual ryegrass (*L. multflorum*). They found that CH_4_ production from forage brassica crops was lower than from annual ryegrass. The conclusion was the same when the emissions were expressed as per unit of incubated organic matter, neutral detergent fiber, digestible organic matter and digestible neutral detergent fiber. Jayanegara et al. (63) and Dillard et al. (64) speculated that the plant secondary metabolites in brassica crops play a role as methanogen inhibitors in the reduction of CH_4_ production. In an *in vitro* study conducted by Durmic et al. (65), a hybrid of kale and turnip (*B. napus* cv. *Winfred*) and turnip produced 30% less CH_4_ in comparison with the control arrowhead clover (*Trifolium vesiculosum*), but there were great variations among different cultivars of the same forage species. Broccoli (*B. oleracea*) and a hybrid of turnip and forage rape (*B. campestris* × *B. napus*) were not significantly different from arrowhead clover in CH_4_ production. The characteristic phenomenon of methanogen inhibition *in vitro* is the release and accumulation of a large amount of hydrogen (66). These studies did not measure the concentration of hydrogen emitted, which makes it difficult to determine if methanogen inhibitors were present in these feedstuffs. Sun and Pacheco (32) did not find a significant difference in methanogenesis between forage brassicas, including kale, turnip, forage rape and swede, and perennial ryegrass in an *in vitro* study, and significant emissions and accumulation of hydrogen were not observed, suggesting that no methanogen inhibitor exists in brassica forages.

Researchers have also used GSLs and their breakdown products directly to test for effects on CH_4_ emission in *in vitro* rumen fermentation studies. The addition of GSLs extracted from mustard cake at doses of 0, 9, 18, 27 and 45 mg/100 mL did not adversely affect the total short-chain fatty acid (SCFA) concentration and microbial activity, but the proportion of CH_4_ in the total gas production increased with the amount dosed, indicating no inhibitory effects (67). Reduced CH_4_ production and hydrogen accumulation were observed when allyl isothiocyanate, a breakdown product of sinigrin, was added at doses of 48 and 96 mg/L (68) or at a dose of 75 mg/L (69) for *in vitro* incubation. Similar results were also obtained in *in vitro* studies with allyl isothiocyanate-containing mustard seeds (70) or mustard cake (71). However, the concentrations of GSL breakdown products in these studies were much higher than those that are present in brassica forages as a sole diet in natural conditions. There was no significant difference in CH_4_ production between broccoli cultivars with a high or low content of GSLs as substrates for *in vitro* culture (65). In an animal study, although CH_4_ emissions were not measured, the ruminal concentrations of SCFAs and the ratio of acetate to propionate did not differ in the rumen of steers fed either high- or low-GSL rapeseed (*Brassica napus* cv *Bridger* and *Dwarf Essex*) forage (72). The effects of SMCO on CH_4_ emissions have not been reported. According to the literature mentioned above, GSLs, SMCO, and their breakdown products are unlikely to be methanogen inhibitors.

## Glucosinolates Might Result in Low Methane Emissions via Animal Physiological Parameters

### Effects of Secondary Metabolites in Brassica Forages on Triiodothyronine

**Figure 3:**
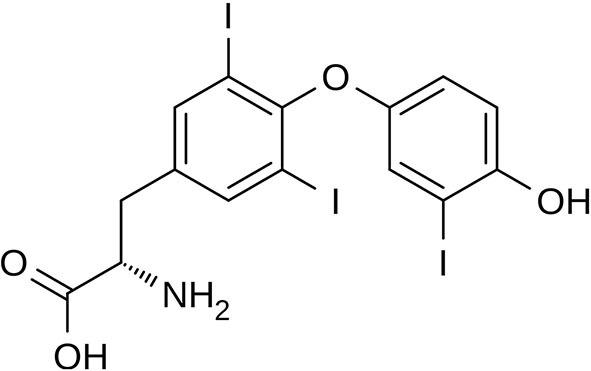
Triiodothyronine structure.

Triiodothyronine (T_3_) (Figure 3) and thyroxine (T_4_) are two thyroid hormones produced and released by the thyroid gland (73). Thyroxine can be converted to T_3_, which is three to four times more metabolically active than T_4_ (74). The thyroid hormones in the blood are mainly present in the form of T_4_ with a ratio of T_4_ to T_3_ at ~14:1. The major fraction of the thyroid hormones is bound with transport proteins, and a small fraction is free and biologically active. Thus, the concentrations of free thyroid hormones, especially free T_3_, are measured as indicators of the hormone activity.

Feeding brassica forages or diets containing GSLs and their breakdown products can affect animal thyroid function and alter thyroid hormone secretion (56, 75–79). For example, feeding turnip (*Brassica rapa* L.) and kale (*B. oleracea* L. var. *acephala* DC) to fattening lambs can increase the concentrations of T_3_ and T_4_ in serum (76). Feeding a diet containing a high content of GSLs to calves resulted in a quadratically increased serum T_4_ concentration, although T_3_ concentration remained within the normal physiological range (79). Diets contained GSLs affect thyroid function in many animal species (56), including pigs (80), mares (81), turkeys (82), hens (83), and turbots (84), suggesting that effects of GSLs on thyroid function are not unique to ruminants, but universal in a wide range of animal species.

The mechanisms of how GSLs affect thyroid hormones in ruminant animals are not clear, but it is believed to be related to iodine and selenium (74). Iodine is a component of the hormones, while deiodinases involved in the conversion of T_4_ to T_3_ are selenium-containing enzymes, and thus selenium is essential for T_3_ production. It is recommended to supplement sheep grazing kale with iodine to lighten the effects of GSLs (75). The impacts of GSLs present in the diet can be counteracted with the supplementation of iodine or iodine plus selenium to sheep (77, 85). Iodine uptake by the thyroid can be inhibited by GSLs and their breakdown products such as goitrin and isothiocyanates (52, 86, 87). In a rat study, nitriles, another group of GSL breakdown products were considered to result in the enlargement of the thyroid (88).

Ruminal microorganisms break SMCO down into dimethyl disulfide, causing anemia (60), but activities on thyroid physiology were not reported. It is unlikely that SMCO has an effect on blood FT_3_ concentration.

### Effects of Free Triiodothyronine on Digesta Retention Time

Thyroid hormones are associated with digesta excretion from the rumen. Sheep exposed to a cold environment (2–5°C) had a 1.5 times greater T_3_ concentration in plasma (152 vs. 62 ng/100 mL) and a 6.2 h shorter rumen mean retention time (11.8 vs. 18 h) than those exposed to a warm environment (22–25°C) (89). A greater T_3_ concentration (103 vs. 21 ng/100 mL) in sheep plasma resulting from a daily injection of 0.25 mg T_3_ also reduced the rumen mean retention time (17.8 vs. 20.4 h) (89). The removal of the thyroid gland from sheep housed at 22–25°C caused T_3_ concentration to drop from 38 ng/100 mL to zero in plasma and the rumen mean retention time extended from 17.9 to 23.6 h (89). In a study with mature ewes by Lourenço et al. (90), it was observed that liquid rumen retention time was 18.5 vs. 26.3 h, while T_3_ concentration was 83–99 vs. 59–67 ng/100 mL at high (25°C) and low (11°C) temperatures, respectively (90). When mature wethers were injected with 300 μg of FT_3_ every 2 days, the blood FT_3_ concentration increased from 16 to 54 ng/100 mL, and digesta retention time in the whole digestive tract was reduced by 4 h (91).

The mechanism of thyroid hormones affecting rumen physiology is unknown, but in the human body, thyroid disorders are associated with gastrointestinal dysfunction (92). It is proposed that thyroid hormones affect gut motility either directly or via a central stimulatory effect on the chemoreceptor trigger zone.

### Effects of Digesta Retention Time on Methane Emissions

Pinares-Patino et al. (93) found that short digesta mean retention time, especially in the particulate phase, was associated with low CH_4_ production in sheep fed alfalfa. In a study on mature ewes divergently selected for high and low CH_4_ yields, a shorter mean retention time of particulate and liquid digesta was associated with low CH_4_ yield (94). This association was further confirmed in a recent study by Bond et al. (95), who measured rumen digesta flow and CH_4_ yield using open-circuit respiration chambers in ewes phenotypically differing in CH_4_ emissions. Using simulation with mathematical models, Huhtanen et al. (96) also demonstrated that dairy cows and sheep with short digesta retention times emit less CH_4_.

Low-CH_4_ yielding sheep not only had smaller rumens (97), but these sheep had different rumen microbial communities compared to high-CH_4_ yielding sheep (98). Detailed studies of these sheep indicated that their microbial communities were fermenting feed using different pathways that led to the observed lower CH_4_ yields (45), and these differences were attributed to faster passage rates through the rumen of the low-CH_4_ yield sheep (45, 99). Furthermore, a recent study integrating rumen wall transcriptome data and CH_4_ phenotypes found that a set of rumen muscle genes is involved in cell junctions, which could be potential regulators of rumen digesta retention time and thus could be a molecular mechanism for the association of rumen digesta retention time with CH_4_ yield in sheep (100).

### Effects of Free Triiodothyronine on Methane Emissions

Elevating serum FT_3_ by intramuscular injection of FT_3_ in sheep can result in shorter digesta retention time and consequently reduce CH_4_ yield by 8% (91). An increase in blood FT_3_ concentration in sheep a result of a decrease in ambient temperature also lead to reduced digesta mean retention time and decreases CH_4_ yield (101).

### Hypothesis

**Figure 4:**
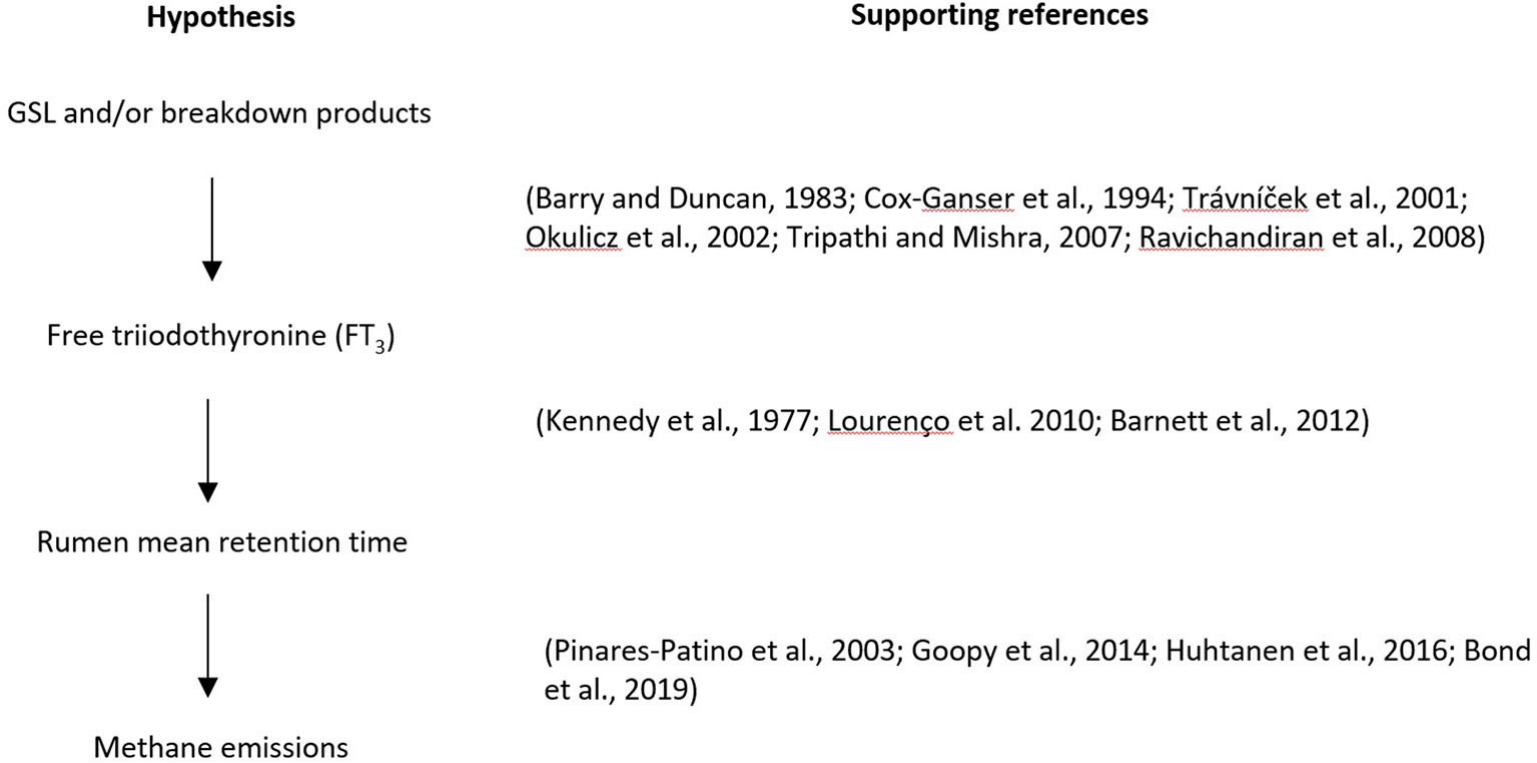
Diagram of the proposed hypothesis and supporting references.

Based on the literature reviewed here, a hypothesis is proposed that under normal farming conditions, the secondary metabolites GSLs and/or their breakdown products in brassica forage crops do not directly inhibit the growth and activity of methanogens, but increase blood FT_3_ concentration in ruminants, resulting in a decrease in digesta mean retention time in the rumen, thereby reducing CH_4_ emissions (Figure 4).

## Concluding Remarks

Climate change is a topic of increasing concern in the world. Anthropogenic activities, including industrial and agricultural production, emit greenhouse gases that are the main drivers of climate change. Methane is an important greenhouse gas, and ruminal fermentation of feed is an important source of CH_4_. Exploring simple, effective and low-cost approaches without side effects to mitigate CH_4_ emissions from ruminants is supported by the governments of most countries. Reducing CH_4_ emissions from ruminants not only helps to slow down climate change but also improves the feed energy efficiency of ruminants. Therefore, the study of the ruminant CH_4_ emission mechanism is of great significance.

This article puts forward a hypothesis that the secondary metabolites of brassica forage crops GSL and its metabolites can elevate the concentration of FT_3_ in ruminants and lead to a reduction in mean ruminal digesta retention time, thereby reducing CH_4_ emissions. This is a new mechanism in which the mitigation of CH_4_ emissions is achieved by manipulating ruminant physiological parameters and goes beyond the existing mechanisms which limit the mitigation to the manipulation of rumen microorganisms and their substrates.

If this hypothesis is confirmed, it will be a new direction for the mitigation of CH_4_ emissions from ruminants and will expand research to a new field with great research value. Further questions to be answered include how individual GSLs affect FT_3_ and CH_4_ emissions differently, what the molecular mechanism of GSLs affecting the function of the thyroid gland and the secretion of FT is, how FT_3_ affect rumen digesta retention time, how FT_3_ affect rumen muscle genes, etc. As brassica forages are common forages, this hypothesis is of great value in ruminant livestock methane abatement studies. Approaches to enhanced mitigation efficiency could be found by a deep understanding of these questions.

## Author Contributions

The author confirms being the sole contributor of this work and has approved it for publication.

## Conflict of Interest

The author declares that the research was conducted in the absence of any commercial or financial relationships that could be construed as a potential conflict of interest.
